# Metabolite Profile Changes in Different Regions of Rat Brain Affected by *Ephedra sinica*

**DOI:** 10.1155/2020/8358039

**Published:** 2020-04-27

**Authors:** Zhou Liao, Shanshan Li, Yun Huang, Xiaoquan Luo, Youbao Zhong, Yanhua Ji, Dan Su, Zhifu Ai

**Affiliations:** College of Pharmacy, Laboratory Animal Science and Technology Center, Jiangxi University of Traditional Chinese Medicine, 1688 Meiling Road, Nanchang 330006, China

## Abstract

*Ephedra sinica* Stapf (EP) has a long medication history dating back centuries in the world. There were some reports of adverse effects in the central nervous system (CNS) resulting from administration of a drug containing EP or ephedrine. Compared with alkaloid monomer compounds, the effects of EP on the CNS are usually neglected. It is necessary to explore CNS affection which is helpful to use EP rationally. However, the affection and the changes of substances by EP in the brain are still unknown because the effects of drug on the brain also exhibit different tendency and distribution and usually lead to diversity of metabolite alteration in different regions. In this study, metabolomics based on different brain regions was used to investigate the affection mechanism of EP in the CNS. The metabolites in 6 brain regions from a rat that underwent oral administration with EP for 14 days were determined by UPLC/Q-TOF-MS. Brain histological examinations showed that there were no obvious lesions in EP administration groups. Partial least square-discriminant analysis (PLS-DA) displayed that there were significant separations between control and EP administration groups. 7 CNS biomarkers were found and identified in different regions. 3 metabolic pathways were disturbed by EP, including amino acid metabolism, phospholipid metabolism, and amino sugar metabolism. Furthermore, all biomarkers were significantly changed in the cortex after administration. This study may be helpful to understand the affection mechanism of EP in the CNS and improve cognition of brain regional characteristics.

## 1. Introduction

*Ephedra sinica* Stapf (EP) has a long medication history dating back centuries in the world [1]. In oriental countries, EP has often been used to treat upper respiratory tract infections (URTIs), headache, general aching, acute glomerulonephritis, and chronic renal failure [2, 3]. In some western countries, it also has been used as herbal supplements and health products [4]. Due to its strong pharmacological activity, EP is often adopted as a monarch drug in Chinese medicine formulae, provides the main therapeutic effect, and plays an indispensable role in treating clinical respiratory diseases [5, 6]. Ascribed to clinical symptoms of URTIs, which usually last for 3–10 days, with a few lingering symptoms such as coughing remaining for a longer period [7], patients usually take EP preparations 3–7 days in traditional Chinese therapy or may be extended for weeks in some cases [8, 9].

The main pharmacological effect of EP is attributed to ephedrine-type alkaloids, such as ephedrine and pseudoephedrine. They are sympathomimetic drugs and also used to treat common cold and asthma in modern chemistry and medicine [1, 10]. However, there were many reports of adverse reactions in the central nervous system (CNS) resulting from longer periods of administration of drug containing EP or ephedrine, and the clinical manifestation varied from dizziness and strokes to seizures [11, 12]. Therefore, ephedrine and total ephedrine were recommended not to exceed the doses of 20 mg and 25 mg per day, respectively, and not suitable for long-term intake [13, 14]. Compared with alkaloid monomer compounds, the effect of EP on the CNS is still neglected. According to the content of ephedrine in EP (about 2%) [15] and the limitation of 20 mg/day, 0.125 g/day for EP could be predicated. In fact, the usage of EP is often far more than 0.125 g, especially in URTIs treatment [16, 17]. Besides, the administration for URTIs is usually more than a week. Therefore, it is necessary to explore the EP effects on the CNS, which is helpful to understand and use EP. However, the effects and the changes of substances in the brain by EP are still unknown.

Metabolites are substances chemically transformed during metabolism in response to a particular stimulus or a disease state, giving the in vivo information about the actual status of the body [18]. Metabolomics has the ability to rapidly assess potential injury and monitor changes during chronic drug administration [19]. The analysis of metabolites in the brain provides a sensitive approach to research potential changes in the CNS, which is difficult to find in histological examinations [20–22]. Brain metabolomics can provide abundant information to acquire the affection evaluation by various endogenous substances differentially expressed in the CNS. Ultraperformance liquid chromatography (UPLC) coupled with mass spectrometry (MS) has been applied widely in metabolomics studies owing to its higher resolution, sensitivity [23], and wider detection of metabolites [24].

The brain is a highly integrated system containing multiple regions, premotor cortex, hypothalamus, striatum, hippocampus, cerebellum, and oblongata, performing different physiological functions and metabolic patterns [25]. Besides, drug effects on the brain also exhibit different tendency and distribution and usually lead to diversity of metabolite alteration in different regions [26]. In our previous research, it was found that alkaloids in EP could rapidly cross the blood-brain barrier of rats after oral administration, presenting the different characteristics of regional distribution in 6 brain regions mentioned above. Especially, the cortex and hippocampus were the main distribution regions of alkaloids [27–29]. However, whether the specific distributions of EP cause the different changes of metabolism in brain regions is still unknown. Thus, metabolomics based on different regions will be meaningful to understand the affection mechanism of EP in the CNS. On this basis, the analysis of metabolite profiles in different brain regions and histological examinations were carried out after 14 days of oral administration with EP in rats. With principal component analysis, we investigated the chromatographic data used to identify the control and the dosed rats based on the differences of their metabolic profiles. Biomarkers associated with CNS effects of EP were determined and identified. Through the detection of the metabolic alteration in different brain regions, the results of this investigation will provide useful information to understand CNS effective mechanism.

## 2. Materials and Methods

### 2.1. Herbal Materials, Chemicals, and Reagents

Dried herbaceous stems of *Ephedra sinica* Stapf (EP) were purchased from Inner Mongolia and authenticated by Head Pharmacist Shuyao Wu (the Jiangxi University of Traditional Chinese Medicine). A voucher specimen (no. 17-10-18-04) has been deposited in the herbarium of the College of Pharmacy, Jiangxi University of Traditional Chinese Medicine. Methanol (HPLC grade) was obtained from Fisher Scientific (Fair Lawn, NJ, USA). Formic acid (for MS, ≥99%) was purchased from Sigma-Aldrich Co., Ltd. (St Louis, MO, USA). All other reagents were of analytical purity. Distilled water was supplied by Watsons Group (Hangzhou, China).

### 2.2. EP Water Extract Preparation

The dried and pulverized plant material of EP (500 g) was refluxed with water of 10 times for 2 h, and then refluxed twice with water of 8 times for 2 h. Then, the three water extracts were combined and concentrated at 50°C by using a rotary evaporator. Finally, two EP water extracts, that is, 1 mL extract containing 1 g or 3 g raw medicines, were obtained for oral gavage. The two EP extracts were stored at 4°C.

### 2.3. Animals and Treatment

All animal experiments were performed in compliance with the ARRIVE guidelines on animal research [30]. All animal experiments were completed in an aseptic processing room supported by the Laboratory Animal Science and Technology Center, Jiangxi University of Traditional Chinese Medicine. All procedures were approved by the Animal Ethics Committee of the institution. All possible measures were taken to minimize the sufferings of the animals throughout the experimental procedures.

24 male Wister rats at postnatal age of 8 weeks (weight 180–220 g) were provided by the Hunan Silaike jingda Laboratory Animal Co., Ltd. (Changsha, P.R. China). All animals were acclimated for at least one week in standard animal houses with regulated temperature (22 ± 2°C) and humidity (50 ± 20%) and a periodic cycle of 10 h light and 14 h darkness. They were freely accessible to common diet and water.

After 7 days of acclimatization, the rats were randomly divided into three groups (*n* = 12/group) as follows: healthy control group (CG), low-dose group (LDG), and high-dose group (HDG). In each group, 6 rats were randomly selected for histological examination and another 6 rats for the brain metabolomics study.

All rats received administration via oral gavages once a day for 14 d. LDG was orally administered with the EP extract (1 mL extract containing 1 g EP raw medicines) at a dose of 15 g EP/kg/day. HDG was orally administered with the EP extract (1 mL extract containing 3 g EP raw medicines) at a dose of 30 g EP/kg/day. The CG was given the equal volume of water as the EP administration group.

### 2.4. Histological Examinations

The rats for histological examinations were sacrificed by cervical dislocation. Their whole brain tissues were collected immediately and washed with phosphate buffer saline (PBS), stored at −80°C for 24 h. After brain tissues were removed by optimal cutting temperature (OCT) frozen gel, tissues were sliced into 5 *μ*m sections by using a freezing microtome. Brain sections were stained with hematoxylin and eosin for electronic microscope examination.

### 2.5. Sample Preparation for Metabolomics Study

The rats for metabolomics study were sacrificed, and then brain tissues were removed immediately. According to anatomical boundaries, the cerebellum, hippocampus, oblongata, striatum, hypothalamus, and cortex were rapidly dissected on the ice bed and weighed, respectively. Four times the volume of cold water with 0.2% formic acid was added into the separated tissues and homogenized by ultrasonication in ice baths. The mixture was drawn (200 *μ*L) and mixed with 0.2% formic acid (200 *μ*L) and acetonitrile (400 *μ*L). Samples were stored for 30 min at 4°C. The supernatant was drawn (200 *μ*L) for the UPLC/Q-TOF-MS analysis immediately to avoid degradation of samples. Moreover, the samples of 6 brain regions were mixed in equal volumes for the UPLC/Q-TOF-MS analysis in order to detect the overall changes of metabolic profile in the brain.

### 2.6. Instrumentation and Conditions

The controls and samples were analyzed using a Triple TOF SYNAPT G2-Si™ system (Waters Corporation, Manchester, UK) coupled to an ACQUITY™ ultraperformance liquid chromatographic system (Waters Corporation, Milford, MA, USA).

The chromatographic separation was conducted using an ACQUITY™ UPLC™ amide column (50 mm × 2.l mm, 1.7 *μ*m, Waters Corporation). The mobile phase consisted of solution A (0.1% formic acid and 20 mM ammonium formate, pH 4) and solution B (acetonitrile). The column temperature was maintained at 35°C. The linear gradient elution program was as follows: 0–10 min, 80–70% B; 10–12 min, 70–68% B; 12–15 min, 68–60% B; 15–16 min, 60–80% B; 16–19 min, 80% B. The flow rate was 0.3 mL/min. The injecting volume of total samples was set at 2 *μ*L.

The mass spectrometer was equipped with electrospray ionization (ESI) interface. Due to the weak response of mass spectrum in a negative ion model, only the signal in a positive ion model was collected in this study. Data profiling of ions *m*/*z* 50 to 1500 Da was collected at a speed of 0.2 s scan time. The following parameter settings were applied: atomizing gas (N_2_) flow, 800 L/h; desolvation gas temperature, 600°C; ion source heater, 120°C; capillary voltage, 3000 V; cone voltage, 40 V; survey scan. Leucine encephalin (*m*/*z* 556.2771; 1 *μ*g/mL) was used as an external reference (LockSpray™) to centroided and mass corrected data. The reference solution was infused to 5 *μ*L/min and sampled continuously at 30 s intervals, generating a reference ion at 556.2771. The data acquisition mode was an MS^E^ mode. The MS^E^ mode comprises two full scan functions, one acquired under low-energy conditions set at 4 V to obtain accurate mass data of intact precursor ions and one acquired under high-energy conditions with a ramp of 20–50 V to obtain product ions and corresponding accurate mass data [31].

### 2.7. Data Analysis

The UPLC/Q-TOF-MS original chromatograms were imported into MarkerLynx software for automatic peak matching, peak alignment, and normalization, and then a data table containing each detected feature (*m*/*z*, retention time) of all samples with their relative intensity were obtained. To visualize the similarities and differences among these data, the data of three groups in each brain regions were imported into Ezinfo 2.0 software for partial least square-discriminant analysis (PLS-DA), respectively. The data were scaled using Pareto scaling, in which the weighting ensures the inclusion of metabolites at low concentrations while minimizing the effects of noise [32]. Meanwhile, score plots and loadings plots were applied to analyze the trends or clustering of the data and searching for potential biomarkers.

GraphPad Prism v.6 software (San Diego, CA, USA) was used for statistical analysis. All values were expressed as mean ± SEM. The *P* value less than 0.05 was considered to be statistically significant.

## 3. Results and Discussion

### 3.1. Validation of Instrument Conditions and Sample Preparation Methods

The instrument conditions and sample preparation methods were validated at first, including the within-day stability, the precision of injection, and the repeatability of sample preparation. Six ions with high abundances covering the whole analysis process were selected for method validation. The retention time and mass pairs were as follows: *m*/*z* 291.0582, 1.86 min; *m*/*z* 162.0939, 3.63 min; *m*/*z* 338.3276, 6.31 min; *m*/*z* 288.1881, 5.68 min; *m*/*z* 476.3287, 8.15 min; *m*/*z* 346.3198, 10.35 min. The relative standard deviations (RSDs) of retention time and peak intensities for the selected ion in brain samples were calculated. The repeatability of sample preparation was evaluated through repeated preparation of five identical samples. The precision of injection was tested by six repeated injections in a sample. To test within-day stability, 6 injections from a sample after preparation were performed in 24 h with an interval of 4 h. The method is usually used for method validation [33, 34].

The RSDs of retention time for stability, precision, and repeatability were to be 0.15–1.04%, 0.20–1.03%, and 0.29–0.98%, while the RSDs of intensity varied within the ranges of 2.46–7.36%, 1.34–5.06%, and 2.17–5.36%. The results indicated that the instrument and methods have good stability, precision, and repeatability, which adapted to analysis of brain metabolites.

### 3.2. Mixed Samples Analysis

**Figure 1:**
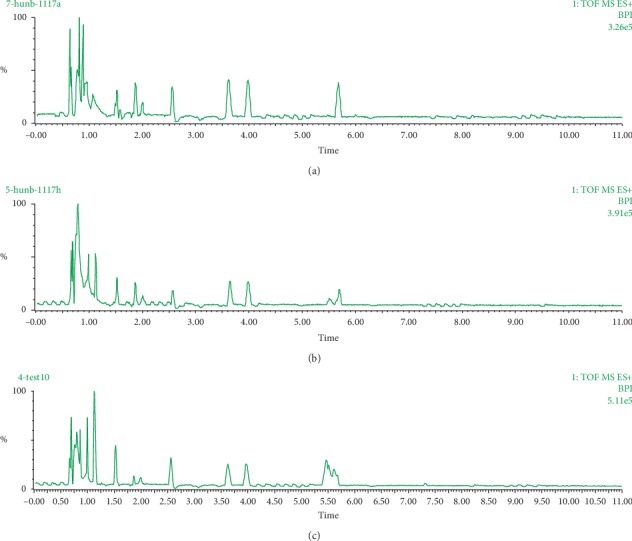
The base peak intensity (BPI) chromatograms of mixed samples of brain tissue in three groups. (a) CG. (b) LDG. (c) HDG.

In order to understand the effects of EP on the brain metabolism pattern from a holistic perspective, we observed the overall metabolic changes of the whole brain before the analysis of 6 brain regions. As shown in Figure 1, the metabolic profiles of EP administration groups were similar to CG, except for the different peak response intensity. The response intensity of the metabolic profile from 3.50 min to 4.50 min in administration groups was weaker than in CG. The response intensity of HDG in 5.00–6.00 min metabolic profile was the strongest among the three groups. The results suggested that the metabolism pattern was disturbed by EP in the rat whole brain.

### 3.3. Identification of Brain Metabolites

**Figure 2:**
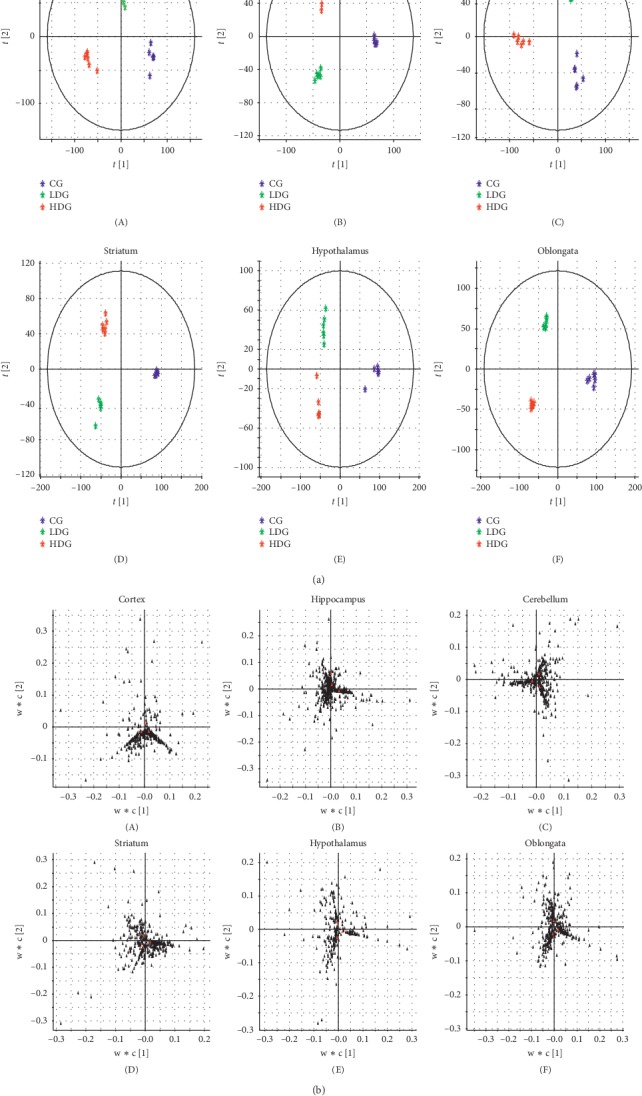
(a) Score plots of different brain regions. Each point represents a sample. Green asterisks represent CG samples, blue asterisks represent LDG samples, and red asterisks represent HDG samples. (b) Loading plots of different brain regions. Each point represents a variable.

Due to the regional specificity of metabolite alteration affected by drugs, we explored the changes of metabolic profiles after administration in 6 brain regions. The UPLC/Q-TOF-MS data of each brain region were evaluated by PLS-DA analysis (Figures 2(a) and 2(b)). Score plots could efficiently distinguish three groups in each brain region. In 6 brain regions, the samples within groups were relatively concentrated, while the boundaries were obvious between three groups. It indicated that the metabolism patterns were changed in each region after administration.

The loading plots were applied to select potential biomarkers by visualizing the influences of variables. As shown in Figure 2(b), the points far from the center were possibly selected as potential biomarkers that interfere with normal metabolism. Finally, some differential metabolites were selected as biomarkers in loading plots of 6 brain regions according to VIP ≧1.5.

These biomarkers were identified subsequently. The information of retention time, accurate molecular weight, and mass fragment was analyzed and checked against with databases, including HMDB (http://www.hmdb.ca/), MassBank (https://massbank.eu/), METLIN (https://metlin.scripps.edu/), and The Golm Metabolome Database (http://gmd.mpimp-golm.mpg.de), and MetabolitePilot™ software (AB Sciex, Canada). Finally, 7 biomarkers were identified (Table 1; Supplementary Figure 1). The biomarker with retention time and *m/z* pairs of 1.00_496.3358 was identified as LysoPC(16:0). The LysoPC(16:0) was taken as an example to illustrate the identification process. In the low-energy spectrum, the corresponding peak was found according to the retention time, and then an accurate mass ([M + H]^+^ at *m*/*z* 496.3358) was obtained. The molecular formula was C_24_H_50_NO_7_P by calculation. Then, the information of biomarker was checked with the databases such as HMDB and Lipid Maps. The compound was preliminarily identified as LysoPC (16 : 0). The structure of the compound was further confirmed by the analysis of fragment ions that were obtained from the high-energy spectrum. The fragment ions *m*/*z* 184.0544 represent [C_5_H_15_NO_4_P]^+^. The identification processes of other biomarkers were similar to LysoPC (16 : 0). Finally, the metabolic pathway analysis revealed that these biomarkers were associated with 3 metabolic pathways, including amino acid metabolism, phospholipid metabolism, and amino sugar metabolism.

### 3.4. Histological Examinations and the Analysis of Biomarkers in Brain

**Figure 3:**
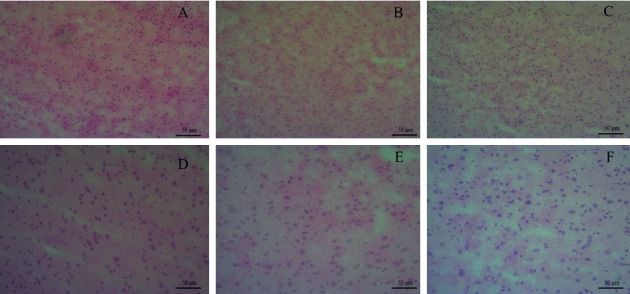
Hematoxylin-eosin staining to delineate the effect of EP on brain histopathologic changes. (a) CG (×100); (b) LDG (×100); (c) HDG (×100); (d) CG (×200); (e) LDG (×200); (f) HDG (×200). Scale bar, 100 *μ*m.

The changes of brain tissues affected by EP were detected via histological examinations (Figure 3). The CG group showed the normal structure of the brain. The obvious lesions were not observed in the brains of administration groups, which suggested that the oral administration of EP did not show the obvious pathological damage. However, the results of the metabolomics study have implied that EP disturbs the normal metabolic pattern in the brain. The interior of the organism attempts to maintain homeostasis by self-regulating and the internal metabolism begins to change, thereby causing no clearly pathological injury in tissue [35]. Therefore, the alterations of metabolism in vivo are generally earlier than the pathological changes.

**Figure 4:**
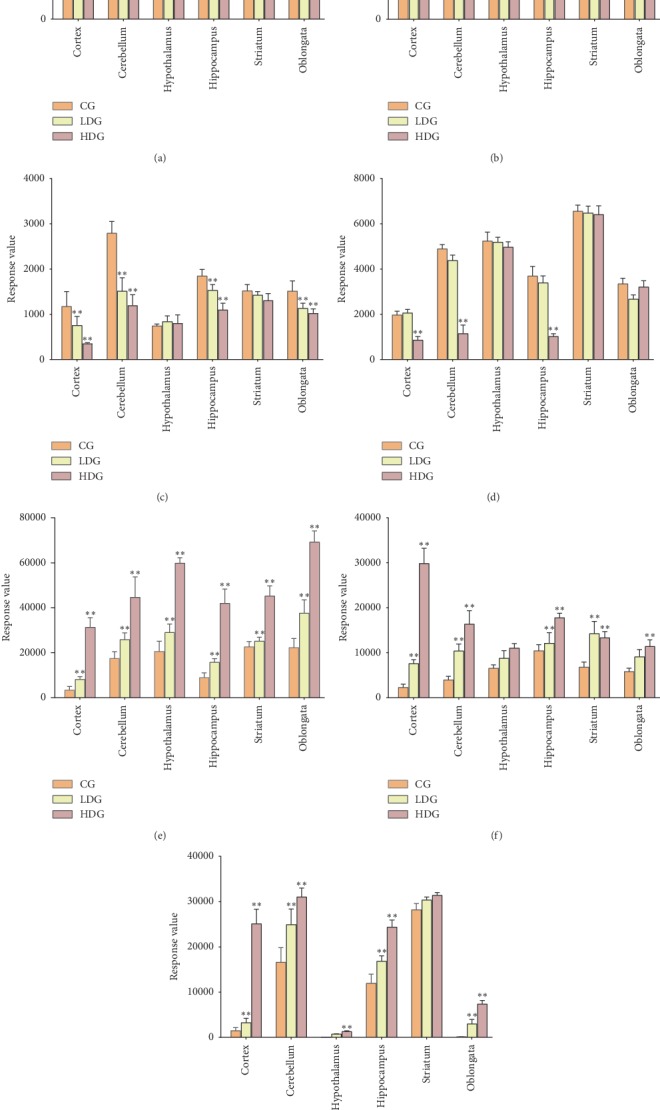
Changes of biomarkers in six brain regions. All data are presented as mean ± SEM (*n* = 6/group); ^*∗*^*P* < 0.05 and ^*∗∗*^*P* < 0.01, compared with the CG. (a) Hydroxylysine. (b) N-Carbamoylsarcosine. (c) Serinyl-valine. (d) Trap-Val-Arg. (e) GPC. (f) LysoPC (16 : 0). (g) Neuraminic acid.

The changes of biomarkers in different regions are shown in Figure 4. Meanwhile, the metabolic pathway analysis showed that three metabolic pathways were disturbed by EP in CNS, including amino acid metabolism, phospholipid metabolism, and amino sugar metabolism.

#### 3.4.1. Amino Acid Metabolism

In this study, some amine acid metabolites were changed in the CNS after administration of EP, including N-carbamoylsarcosine, hydroxylysine, serinyl-valine, and tryptophan-valine-arginine (Trap-Val-Arg). Amino acids are the essential components of protein in the cell and also participate in the neuronal signal transmission and multiple metabolic pathways in the CNS. Amino acid metabolism disorder causes dysfunction of the brain and is associated with epilepsy, stroke, and other neurological diseases [36]. Interestingly, these biomarkers related to amino acid metabolism were significantly decreased in the cortex after administration, while they had no obvious changes in the hypothalamus. It suggested that EP changed the amino acid metabolism and reduced the levels of amino acids in the cortex.

**Figure 5:**
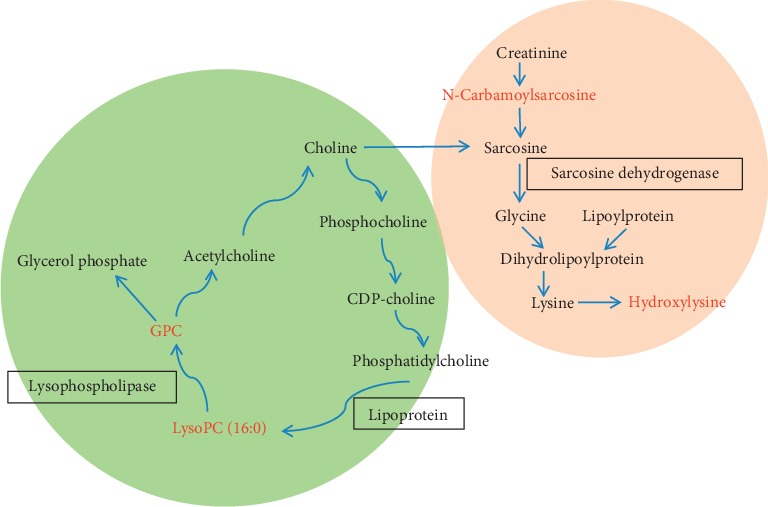
Metabolic pathway related to biomarkers. Metabolites highlighted in red are biomarkers. Metabolites in the green area participate in phospholipid metabolism. Metabolites in the red area participate in amino acid metabolism.

N-Carbamoylsarcosine is deaminated to sarcosine by N-carbamoylsarcosine amidohydrolase. Sarcosine is rapidly hydrolyzed to glycine by sarcosine dehydrogenase (Figure 5). Glycine as an inhibitory neurotransmitter acts on receptors coupled to chloride channels and leads to hyperpolarization in the postsynaptic membrane and inhibition of neuronal excitability [37]. In this study, the significant downregulation of N-carbamoylsarcosine was observed in the cortex and striatum after administration of EP. Interestingly, the change in the cortex was more obvious than in the striatum. In the cortex, the concentration of the HDG was decreased by about 80% compared with the CG (Figure 4). As the downstream metabolites of N-carbamoylsarcosine, the level of glycine would be decreased in the brain, especially in the cortex. The decline of glycine causes the uncontrol of postsynaptic membrane excitability, resulting in the neuronal excitation [37, 38], which may be associated with CNS excitability induced by EP.

Hydroxylysine is a hydroxylated derivative of lysine (Figure 5). Lysine, as an essential amino acid, has the functions of protecting the nervous system and promoting growth and development [39]. Compared with the CG, the level of hydroxylysine was significantly decreased in the cortex and striatum after administration (Figure 4), which indicated the downregulation of lysine in the brain affected by EP. The decrease of lysine leads to some psychiatric diseases, such as epilepsy [39], which may be related to the mechanism of epilepsy induced by EP.

In this study, we found that some polypeptide biomarkers were changed, including serinyl-valine and Trap-Val-Arg. These polypeptides are intermediate products of protein decomposition and would enter specific amino acid metabolic pathways through further proteolysis. Some polypeptides have physiological or cell-signaling effects. Serinyl-valine is a dipeptide composed of serine and valine, while Trap-Val-Arg consists of tryptophan, valine, and arginine. Serine, abundant in the brain, participates in several physiological processes, such as neurotransmission, synaptic plasticity, learning, and memory [40]. Valine, as a branched chain amino acid, is critical to human, which involved in stress and energy. The deficiency of valine in the brain would cause neurological defects, mental retardation, and ataxia [36]. Tryptophan, as the precursor of both serotonin and melatonin, is associated with sleep, wakefulness, and emotion [41, 42]. Overall, these amino acids have unique physiological functions and activities in the brain.

In this study, the level of serinyl-valine was generally decreased in several brain regions after administration. In the cortex, cerebellum, and hippocampus, the Trap-Val-Arg was significantly decreased in the HDG compared with the CG (Figure 4). These results suggested that amino acid metabolism was interfered by EP in several brain regions, which may influence the normal physiological function of the CNS.

#### 3.4.2. Phospholipid Metabolism

In this study, some phospholipids were significantly increased in 6 brain regions after administration, including glycerophosphocholine (GPC) and LysoPC (16 : 0). The disorder of phospholipid metabolism interferes with the normal physiological function of the brain [43]. GPC, as the major form of choline storage in the cytosol, could be converted into acetylcholine (Ach) (Figure 5). In this study, the significant increase of GPC in each brain region was observed in administration groups compared with the CG (Figure 4), which indicated that the levels of Ach and choline were changed induced by EP in the rat brain. Ach is widely distributed in the brain and has unique physiological function in different brain regions. In the hippocampus, Ach participates in the formation of memory. It is also associated with the mechanisms of reward and addiction in the striatum. In the cortex, the change of Ach is closely related to the level of attention [44]. The changes of Ach in several brain regions induced by EP may lead to the alteration of multiple physiological functions in the CNS. The results implied that the effect on mechanisms of EP in the brain may be associated with the increased level of Ach.

LysoPC (16 : 0), as a lysophospholipid, can be metabolized to GPC by lysophospholipase (Figure 5). Compared with the CG, the level of LysoPC was significantly increased in 6 brain regions in a dose-dependent manner (Figure 4). The change was most obvious in the cortex among 6 regions. The high level of LysoPC causes the hemolytic symptoms and injures nerve cells [45].

#### 3.4.3. Amino Sugar Metabolism

Amino sugar metabolism was also disturbed by EP. Neuraminic acid is a common sialic acid in human tissues and often N-acylated at the C-5 position. Sialic acid, as a class amino sugar, is usually located at the end of the sugar chain on the cell membrane. In the CNS, neuraminic acid mainly combines with sphingolipid to form ganglioside, which plays an important role in neurogenesis, synaptic plasticity, and nerve impulse transmission [46]. Compared with CG, the concentration of neuraminic acid was significantly increased in 6 brain regions after administration, especially in the cortex, hypothalamus, and oblongata (Figure 4), which suggested that EP markedly changed the amino sugar metabolism in the three regions. Moreover, it is worth noticing that sialic acid has a negative center, which causes neuronal excitation through producing a rapid succession of action potentials [47]. It may be related to the CNS excitation induced by EP.

### 3.5. Analysis of Biomarkers in 6 Brain Regions

To explore the effects of EP in different brain regions, the changes of biomarkers in different regions were analyzed. Interestingly, it is found that all biomarkers were significantly changed in the cortex after administration of EP. Although the changes of some biomarkers were obvious in some regions, there was no region where all biomarkers were significantly changed after administration, except in the cortex (Figure 4). After administration, the overall changes of biomarkers in the cortex were more significant than in other regions, especially neuraminic acid, GPC, and LysoPC (16 : 0). In our previous study, the cortex was also the main distribution region of the ephedrine-type alkaloids in the rat brain after oral administration with EP [27]. These results suggested that the specific distributions of EP and ephedrine-type alkaloids may cause the regional specificity of metabolic changes in the brain. Meanwhile, the cortex was an important affected region by EP in the brain. The cortex participates in several organism functions, such as bodily awareness, feelings, emotions, and regulation of the central autonomic nervous system. It plays an indispensable role in maintaining normal physiological function [48, 49]. The changes of the cortex affect the functions of the CNS and trigger a variety of diseases, such as stroke [49]. However, EP changed the metabolic patterns in the cortex, which may lead to the brain function dysfunction and adverse reactions. In this study, we found that the alkaloids of EP could rapidly cross BBB and enter into the brain after administration and are mainly distributed in the cortex and disturb the normal metabolism of the cortex, which may lead to CNS adverse reactions.

## 4. Conclusion

In conclusion, this work demonstrated the CNS effects of EP in metabolite profiles. The different changes of metabolites were observed in 6 brain regions after the administration of EP. 7 biomarkers were found and identified in different brain regions through the PLC-DA analysis. The metabolic pathway analysis revealed that 3 metabolic pathways were affected by EP, including amino acid metabolism, phospholipid metabolism, and amino sugar metabolism. Furthermore, we found that the cortex is the major influenced area affected by EP in the brain, in which all biomarkers were significantly changed after administration. This study may improve cognition of CNS regional characteristics and promote development of its mechanism research affected by EP.

## Figures and Tables

**Table 1:** Potential biomarkers identified in the positive mode.

Number	Retention time (min)	Biomarker	Molecular formula	Parent ion (*m*/*z*)	Fragment ion (*m*/*z*)	Error (ppm)
1	3.97	N-Carbamoylsarcosine	C_4_H_8_N_2_O_3_	133.0612[M+H]^+^	114.0471, 90.0358	3
2	3.62	Hydroxylysine	C_6_H_14_N_2_O_3_	163.1078[M+H]^+^	144.9638	1
3	2.56	Serinyl-valine	C_8_H_16_N_2_O_4_	205.1182[M+H]^+^	118.0668, 60.0360	−0.5
4	0.70	Trap-Val-Arg	C_22_H_33_N_7_O_4_	460.2637[M+H]^+^	415.2028	−3
5	5.46	GPC	C_8_H_20_NO_6_P	258.1102[M+H]^+^	221.0004	0.4
6	1.00	LysoPC (16 : 0)	C_24_H_50_NO_7_P	496.3358[M+H]^+^	184.0544	−8
7	1.53	Neuraminic acid	C_9_H_17_NO_8_	268.1035[M+H]^+^	136.0425	3

## Data Availability

The data used to support the results of this study are available from the corresponding author upon request.

## Supplementary Materials

Supplementary Materials Figure S1: the chemical structures and product ions of all biomarkers.
