# Supplementary material for: Metabolite Profile Changes in Different Regions of Rat Brain Affected by Ephedra sinica
Source: Evid Based Complement Alternat Med. 2020 Apr 27;2020:8358039. doi: 10.1155/2020/8358039 (PMC7201840; doi:10.1155/2020/8358039)
Supplement: Supplementary Materials — Figure S1: the chemical structures and product ions of all biomarkers. [file 8358039.f1.pdf]

# Metabolites Profiles changes in different regions of rat brain affected by *Ephedra sinica*

Zhou Liao, Shanshan Li, Yun Huang, Xiaoquan Luo, Youbao Zhong, Ynahua Ji, Dan Su, Zhifu Ai*

*College of Pharmacy, Laboratory Animal Science and Technology Center, Jiangxi University of Traditional Chinese Medicine, 1688 Meiling Road, Nanchang 330006, China.*

* Corresponding author: Tel/Fax: +86 791 87802135. E-mail: aizhifu1668@163.com (Z. Ai).

a

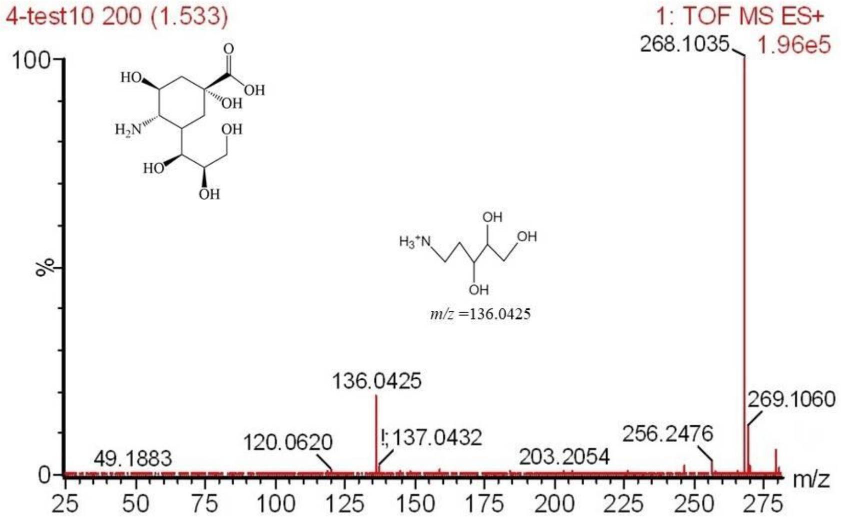
4-test10 200 (1.533)
1: TOF MS ES+
268.1035
1.96e5
100
%
0
$H_3^+N$
OH
$m/z$ =136.0425
136.0425
137.0432
120.0620
49.1883
203.2054
256.2476
269.1060
m/z
25 50 75 100 125 150 175 200 225 250 275

b

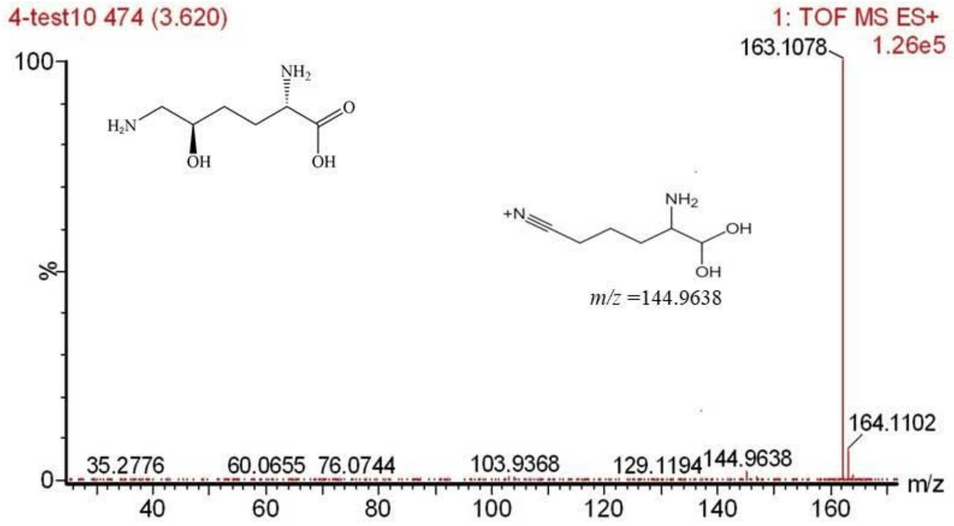
4-test10 474 (3.620)
1: TOF MS ES+
163.1078
1.26e5
100
%
0
$NH_2$
$H_2N$
OH
O
$^+N$
$m/z$ =144.9638
35.2776
60.0655
76.0744
103.9368
129.1194
144.9638
164.1102
m/z
40 60 80 100 120 140 160

c

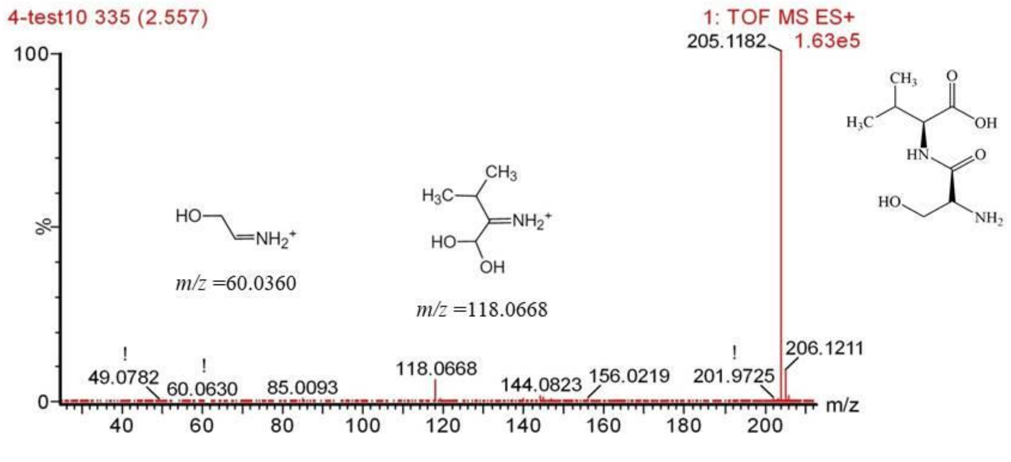
4-test10 335 (2.557)
1: TOF MS ES+
205.1182
1.63e5
100
%
0
HO
$NH_2^+$
$m/z$ =60.0360
$CH_3$
$H_3C$
HO
OH
$m/z$ =118.0668
$H_3C$
OH
HN
O
$NH_2$
49.0782
60.0630
85.0093
118.0668
144.0823
156.0219
201.9725
206.1211
m/z
40 60 80 100 120 140 160 180 200

d

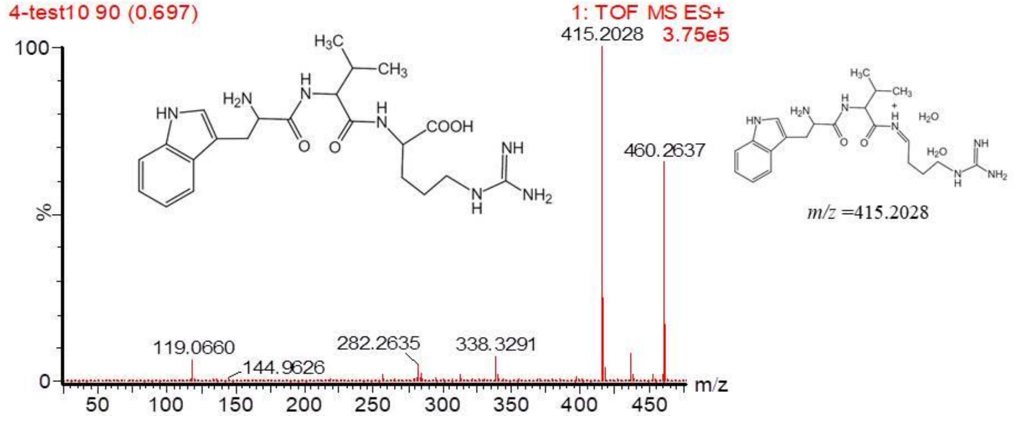
4-test10 90 (0.697)
1: TOF MS ES+
415.2028 3.75e5
460.2637
119.0660
144.9626
282.2635
338.3291
100
%
0
m/z
50 100 150 200 250 300 350 400 450
m/z =415.2028

e

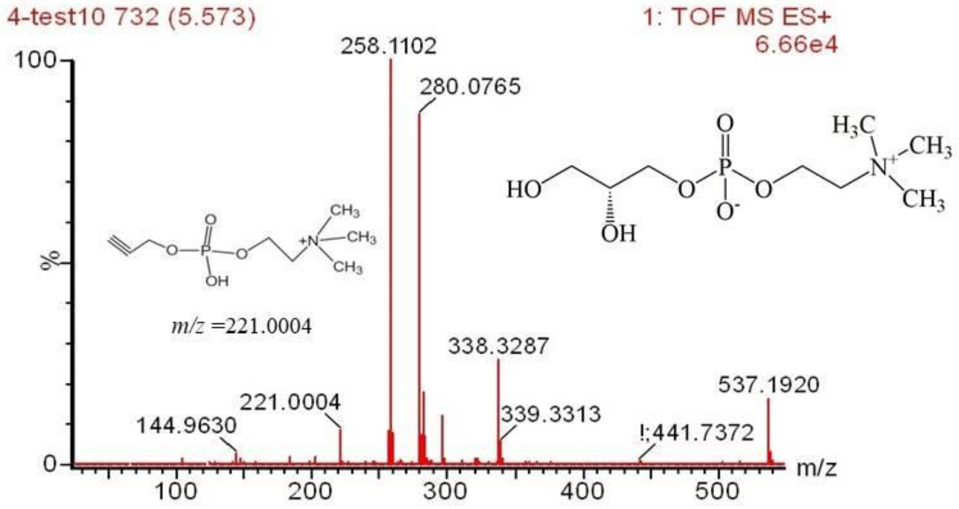
4-test10 732 (5.573)
1: TOF MS ES+
6.66e4
258.1102
280.0765
338.3287
339.3313
537.1920
221.0004
144.9630
441.7372
100
%
0
m/z
100 200 300 400 500
m/z =221.0004

f

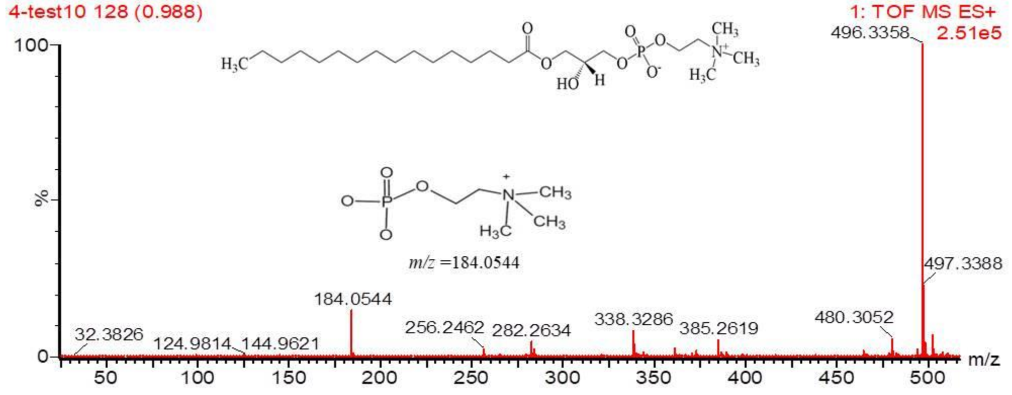
4-test10 128 (0.988)
1: TOF MS ES+
496.3358
2.51e5
497.3388
184.0544
32.3826
124.9814
144.9621
256.2462
282.2634
338.3286
385.2619
480.3052
100
%
0
m/z
50 100 150 200 250 300 350 400 450 500
m/z =184.0544

g

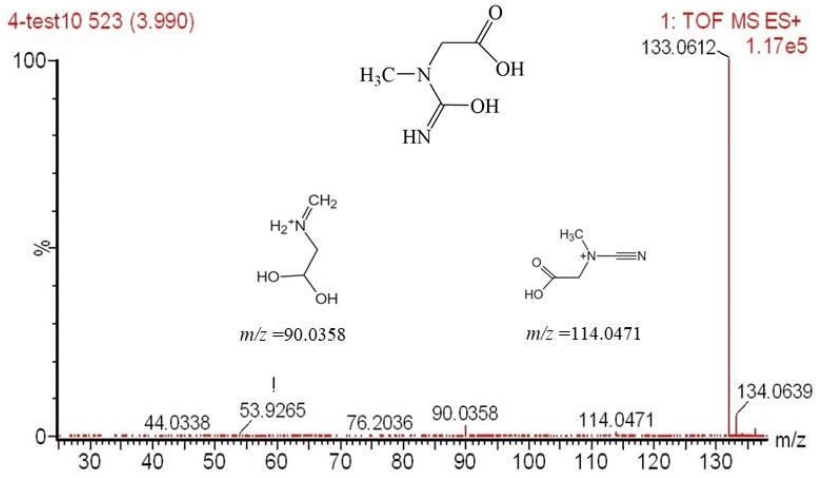


**Fig. S1.** Chemical structures and product ions of Neuraminic acid (a), Hydroxylysine (b), Serinyl-valine (c), Trap-Val-Arg (d), GPC (e), LysoPC(16:0) (f), N-carbamoylsarcosine (g).
